# Using Artificial Intelligence in Fungal Lung Disease: CPA CT Imaging as an Example

**DOI:** 10.1007/s11046-021-00546-0

**Published:** 2021-04-11

**Authors:** Elsa Angelini, Anand Shah

**Affiliations:** 1 NIHR Imperial Biomedical Research Centre, ITMAT Data Science Group, Imperial College London, London, UK; 2 Department of Metabolism-Digestion-Reproduction, Imperial College London, London, UK; 3 Respiratory Medicine, Royal Brompton and Harefield NHS Foundation Trust, London, UK; 4 MRC Centre of Global Infectious Disease Analysis, Department of Infectious Disease Epidemiology, School of Public Health, Imperial College London, London, UK

**Keywords:** Chronic pulmonary aspergillosis (CPA), CT imaging, Artificial intelligence (AI)

## Abstract

This positioning paper aims to discuss current challenges and opportunities for artificial intelligence (AI) in fungal lung disease, with a focus on chronic pulmonary aspergillosis and some supporting proof-of-concept results using lung imaging. Given the high uncertainty in fungal infection diagnosis and analyzing treatment response, AI could potentially have an impactful role; however, developing imaging-based machine learning raises several specific challenges. We discuss recommendations to engage the medical community in essential first steps towards fungal infection AI with gathering dedicated imaging registries, linking with non-imaging data and harmonizing image-finding annotations.

## Introduction

Relatively few fungal species can infect humans; however, certain fungi can cause life-threatening systemic infections in susceptible patient populations. *Aspergillus* species are ubiquitous, saprophytic fungi with airborne conidia that grow on organic matter. *Aspergillus fumigatus* is the principal causative agent of human aspergillosis, which can range from allergy to invasive aspergillosis (IA), a life-threatening infection in immunocompromised hosts, with a further cohort of susceptible individuals developing chronic pulmonary aspergillosis [1] (CPA). Current estimates indicate ~ 63,000 patients develop IA annually within European intensive care units [2]. The age-adjusted number of hospitalizations with invasive aspergillosis and associated mortality and cost has been shown to have increased over a 10 year period in the USA using ICD-9-CM codes with 35,968 cases in 2004 rising to 51,870 in 2013, a 44.2% overall increase [3, 4]. CPA is additionally an increasingly recognized entity in patients without the classical risk factors for invasive fungal disease. It is more prevalent in those with underling lung disease such as previously treated tuberculosis (TB) and COPD, and due to the numbers of people with these conditions, the global burden of CPA is significant, particularly in low-resource settings. Global estimates of CPA are ~ 3 million cases; however, again there is little national prevalence data to be precise [5].

Individuals with both invasive and chronic pulmonary aspergillosis unfortunately have significant morbidity and high mortality. In part, this relates to challenges in diagnosis, difficulty in distinguishing fungal infection from other infections/illnesses and detecting breakthrough infection or treatment failure [6]. Diagnosis is often relied upon through a combination of clinical demographics indicating susceptibility, microbiological tests, imaging and response to antifungal treatment. This complex process fraught with subjectivity is thought to lead to ‘delayed’ or ‘missed’ diagnoses and late initiation of appropriate therapy or change in the event of treatment failure resulting in adverse outcome [7]. In particular, assessing treatment response is challenging and often based on symptomatology and radiology.

Within clinical medicine, over the last decade there has been significant increased interest in the application of machine learning and artificial intelligence (AI) for complex decision making including diagnosis (e.g., medical imaging, pathology, etc.) and guiding therapeutic decision making (e.g., sepsis [8]).

## Imaging AI for Fungal Lung Infection

Regarding in vivo medical imaging, AI is transforming radiological diagnosis with mature developments related to oncology in breast [9] and chest [10]. But such approaches have mostly focused on detecting well-focused lesions such as nodules, relying on manual expert annotation on images for the supervised training. As an alternative, weakly supervised methods have been developed, using image-level or regional-level annotation of the presence or absence of a lesion, without stating where it is. AI in lung imaging is blooming for detection of visual patterns of various lung diseases [11] and commercial imaging AI products have started to emerge such as for TB [12] or COVID19 [13].

Computed tomography (CT) represents the imaging modality of choice for lung infection diagnosis. AI for lung CT imaging bears some specific risks and challenges due to the lack of image standardization and multiple sources of variability (scanner type, acquisition protocol, slice thickness). However, radiological societies are working toward reporting standards for AI tools [14–17] that include demonstration of robustness on external cohorts, similar to previous efforts such as the TRIPOD reporting system for predictive models [18].

## Proof of Concept of Imaging AI on CPA

CT enables localization of CPA-related pathological signs [19] such as pleural thickening, cavities and fungal balls, while chest radiograph imaging, less accurate and with worse inter-reader agreement than CT, is useful only for its negative predictive value [20]. Although diagnosis through CT is not usually difficult, inferring prognosis and radiological progression can be problematic. AI for CPA imaging, however, raises further challenges, as lesions are infiltrating and multiform [21], making contouring unsuitable. Therefore, one might prefer to target lesion *detection* with predictive value for inference of CPA severity or mortality within a time frame rather than mimicking expert precise *contouring*.

**Fig. 1:**
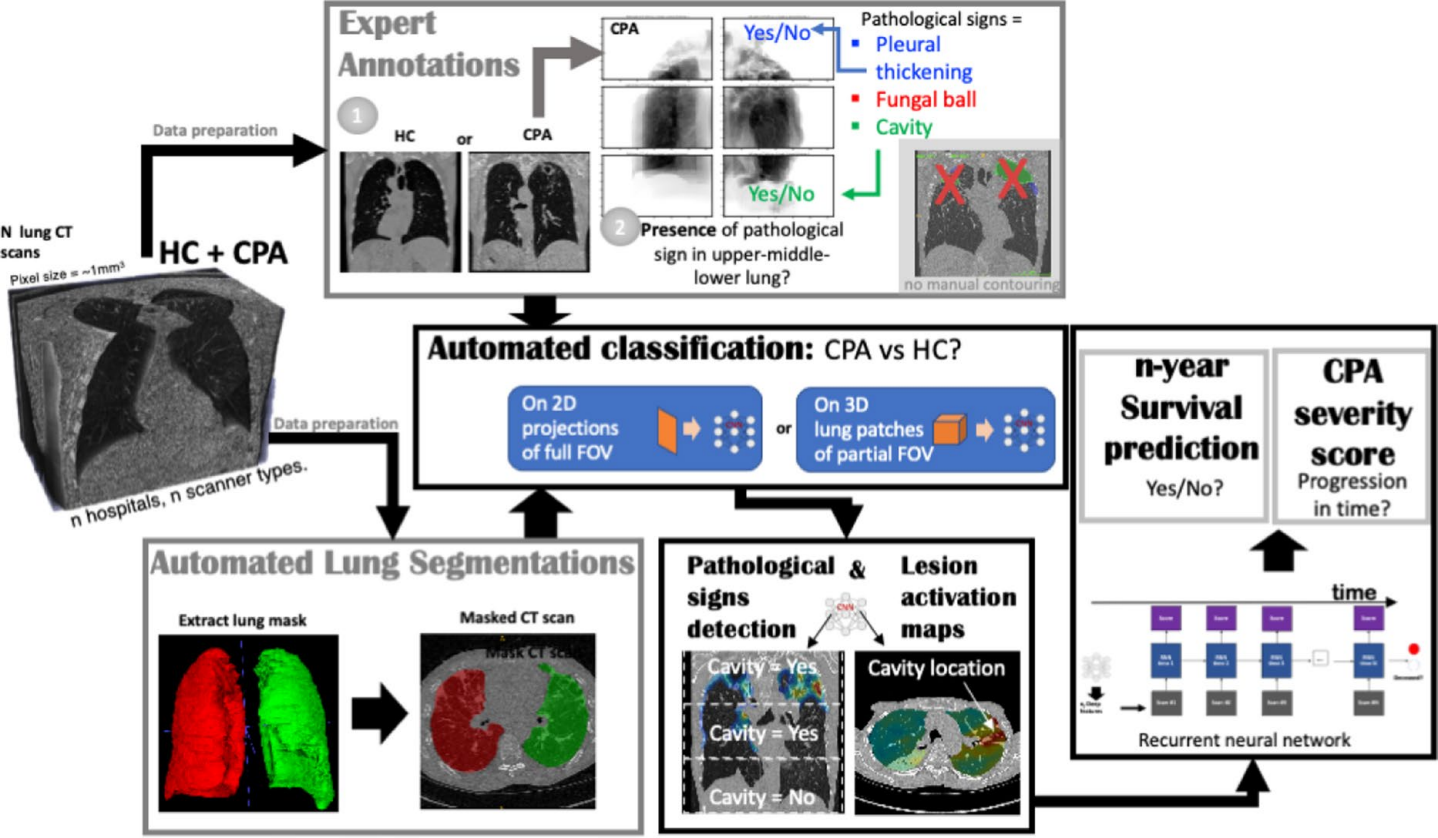
Example of a deep-learning pipeline on CPA lung CT images: equipped with adequate data preparation on a cohort of CT scans (region-level annotation of the presence of CPA pathological signs and lung segmentation), we were able to train deep-learning networks on multiple tasks, from automated binary classification of CPA versus HC, to automated detection of the presence of pathological signs in sub-regions, and further survival prediction within 2 to 5 years with CPA disease severity scoring in time. (HC = healthy control with respect to lung health status), FOV = field of view of the CT scan, CNN = convolutional neuronal network)

We have shown [22, 23] (Fig. 1) that a weakly supervised deep-learning framework is capable of detecting the presence of 3 types of CPA pathological signs with very high accuracy and specificity [23] and can potentially predict mortality within 5 years with high precision [22]. This presents a proof of concept that fungal lung infection may present a unique opportunity for the application of imaging AI to improve outcome.

## Further Recommendations

Although quantitative scoring algorithms have been developed, further work is needed in defining a standard protocol for reporting radiological findings on CPA lung lesions. This is important to refine future annotation task and to benchmark new imaging AI tool against human radiological scoring [24]. Given that other lung diseases typically co-exist in CPA subjects (e.g., cystic fibrosis, emphysema, bronchiectasis), we might also gain in precision and robustness by feeding such information to the network or even specializing the imaging AI tool on sub-cohorts.

To progress the field, there is a need to develop a dedicated imaging cohort with a critical size, large diversity (i.e., aggregation of independent cohorts), and linked with diagnostic data (quantitative symptom scores, serology, microbiology), and treatment information as possible in international registry datasets (e.g., CPAnet) [25]. For initial diagnosis, given the presence of other conditions that can ‘mimic’ CPA (e.g., tuberculosis), the possibility to link to microbiological (including micro- and mycobiome) and serological datasets is likely to be particularly valuable to enhance training and accuracy of machine learning. Non-imaging diagnostic information is typically used as external markers to validate against and to demonstrate the added clinical value of imaging AI (beyond automation of radiological reads). It can also be critical to understand errors made by the imaging AI.

For an imaging cohort to be used in AI, it requires some annotation, and here, a concerted community effort will be required from a number of specialists (e.g., radiology, microbiology, infectious disease, respiratory, etc.) to agree on the ground-truth expert source (e.g., imaging, companion diagnostic data, treatment response) and the granularity of the image annotation (e.g., expert contouring, radiological global read of lesions). Building large imaging cohorts remains tedious but can have very clear impact for fungal lung disease which currently suffers from two challenges: (1) current diagnostic uncertainty and need for subjective amalgamation of multiple observations with high levels of uncertainty; (2) difficulty in determining treatment response because of challenges in monitoring effects using quantitative radiological markers.

Further integration with other omics (genomics, transcriptomics, proteomics) and electronic health record data [26–29] is also a natural progression of AI investigation to understand risk factors for susceptibility, progression or treatment failure. But such cohort of data is costly to acquire and AI on joint imaging-omics healthcare data is still in infancy, due in particular to the challenging identification of relevant relationships among biological entities.

In summary, the application of machine learning and AI presents a unique opportunity within fungal lung infection with the potential to improve diagnosis through early detection, standardization of imaging markers and their quantitative longitudinal monitoring [30], to improve phenotyping through patient stratification and generation of explainable hypotheses, and finally to improve outcome.
